# Overview on Strategies and Assays for Antibiotic Discovery

**DOI:** 10.3390/ph15101302

**Published:** 2022-10-21

**Authors:** Anika Rütten, Teresa Kirchner, Ewa Maria Musiol-Kroll

**Affiliations:** 1 Interfaculty Institute of Microbiology and Infection Medicine (IMIT), Microbiology/Biotechnology, University of Tübingen, Auf der Morgenstelle 28, 72076 Tübingen, Germany; 2 Cluster of Excellence ‘Controlling Microbes to Fight Infections’ (CMFI), University of Tübingen, Auf der Morgenstelle 28, 72076 Tübingen, Germany

**Keywords:** assays, screening, antibiotics, actinomycetes, pathogens

## Abstract

The increase in antibiotic resistance poses a major threat to global health. Actinomycetes, the Gram-positive bacteria of the order *Actinomycetales*, are fertile producers of bioactive secondary metabolites, including antibiotics. Nearly two-thirds of antibiotics that are used for the treatment of bacterial infections were originally isolated from actinomycetes strains belonging to the genus *Streptomyces*. This emphasizes the importance of actinomycetes in antibiotic discovery. However, the identification of a new antimicrobial compound and the exploration of its mode of action are very challenging tasks. Therefore, different approaches that enable the “detection” of an antibiotic and the characterization of the mechanisms leading to the biological activity are indispensable. Beyond bioinformatics tools facilitating the identification of biosynthetic gene clusters (BGCs), whole cell-screenings—in which cells are exposed to actinomycete-derived compounds—are a common strategy applied at the very early stage in antibiotic drug development. More recently, target-based approaches have been established. In this case, the drug candidates were tested for interactions with usually validated targets. This review focuses on the bioactivity-based screening methods and provides the readers with an overview on the most relevant assays for the identification of antibiotic activity and investigation of mechanisms of action. Moreover, the article includes examples of the successful application of these methods and suggestions for improvement.

## 1. Introduction

The rapid spread of multidrug-resistant pathogens is alarming [1]. Consequently, antimicrobial resistance (AMR) was put on the top ten list of global public health threats facing humanity. As the ordinary treatments with the available antibiotics are ineffective, finding and developing new agents to combat infections caused by bacterial pathogens has become an urgent necessity. Many of the antibiotics which are currently applied in human and veterinary medicine were originally isolated from actinomycetes (bacterial strains of the order *Actinomycetales*) [2]. Thus, actinomycetes are an excellent source of antibiotics and other bioactive secondary metabolites. In most cases, the genes encoding the proteins for the production, resistance, transport, etc. of the secondary metabolites, including antibiotics, cluster in the genome (biosynthetic gene clusters (BGCs)). Based on the knowledge obtained from the validated BGCs and the corresponding sequences of protein family domains (Pfams), databases—and subsequently, bioinformatics tools—were established for the mining of the microbial genomes. Such tools make the in silico identification of BGCs possible. A single actinomycete-genome contains more than twenty BGCs. As the output of a cluster analysis usually provides the users with an overview on the hits and their similarity to characterized BGCs, it allows the exclusion of known BGCs, prioritization and/or the selection of potential new BGCs that might deliver a novel compound. In addition to the genome miming tools, traditional methods including strain cultivation, the direct screening of culture supernatants or extracts for biological activity, and finally, compound isolation, which is required for any of the downstream steps of the drug development, are applied in the field. While diverse genome mining tools were described in other reviews [3,4,5,6,7,8], in this article, we emphasize the bioactivity-based screening methods. 

In the first part, we present assays that rely on the diffusion principle and growth inhibition of indicator strains (test strains). The most popular are relatively simple agar diffusion assays and thin-layer chromatography (TLC)–bioautographies. Agar diffusion assays play an important role in diagnostics where they are applied for antimicrobial susceptibility testing with pure substances to identify the appropriate antibiotics for a more targeted treatment. In the drug discovery field, they are particularly useful for the primary screenings of materials from natural producers such as actinomycetes (e.g., culture supernatants, extracts). The primary screenings are usually followed by secondary, more specific screenings that enable the further characterization of the compound. An important part of it is the elucidation of the mode of action. Therefore, diverse target-based assays are utilized, which we describe in the second part of this review. As complex, non-purified samples contain a mixture of metabolites which might interfere with the applied system and lead to unreliable results (e.g., off-target effects), the isolation or at least an enrichment of the active compounds is necessary. The available technologies nowadays make the purification easier [9,10,11,12,13]. In many cases, it is possible to obtain the required quantities and quality for studying the mechanism of action (MOA) and other features of the compound.

In the following sections, we introduce the reader into both diffusion- and target-based screening methods. We describe the advantages and disadvantages of the presented assays and give advice for the optimization of some of the procedures. 

## 2. Primary Screening: Diffusion-Based Assays

The traditional antibiotic discovery workflow starts with the isolation of the producers (e.g., actinomycetes) from different ecosystems. Mainly soil samples are extracted and serial dilutions are streaked on selective solid media for the semi-selection of the microorganisms. After the morphological evaluation of the plates, the promising candidates (e.g., actinomycetes-like colonies) are picked and propagated, typically using series of different media to explore the biosynthetic potential of the isolates. For actinomycetes, agar with the mycelium, culture supernatants as well as extracts are tested in agar diffusion assays.

### 2.1. Agar Diffusion Methods

The first agar diffusion test for the detection of antibiotic activity was developed by Alexander Fleming (1929) after he noticed that around a mould colony (later assigned to *Penicillium notatum*), there was no growth of *Staphylococcus* that was streaked on the same agar plate. Based on this observation, he partially removed a strip of agar from a Petri dish, resulting in a ditch, and filled it with a test solution (medium containing penicillin). He used the other part of the agar plate for the streaking lanes of diverse strains across the ditch (similar to the cross-streak method, where instead of penicillin, potential producers of antibiotics are streaked on the plate). The more susceptible the strain was, the less growth was visible in the proximity of the ditch with penicillin [14,15,16]. Almost one century later, the same basic principle was applied in diagnostics (the determination of the susceptibility of bacterial isolates) and drug discovery (screening of biological material (e.g., extracts) and purified antibiotic candidates). However, several modifications of the agar diffusion test (also referred to as disk diffusion test, Kirby–Bauer test, disc-diffusion antibiotic susceptibility test, disc-diffusion antibiotic sensitivity test) have emerged over the years. These include, for example, the “direct” agar diffusion assay, the agar plug-based diffusion assay, the agar hole-based diffusion assay (well diffusion), the agar disc diffusion assay and bioautographies (Table 1). In the following section, we introduce the basics of each of the methods and compare them with each other.

The “direct” agar diffusion assay is primary used to screen potential antibiotic producers such as actinomycetes [18,19] by their direct exposure to an indicator strain (also called test strain) on solid medium. First, the producer is spotted and incubated at optimal conditions (for actinomycetes, 28–37 °C, 5–14 days). Thereafter, the agar plate is overlaid with a suspension of the indicator strain (the optical density of the suspension strongly depends on the protocols found in the literature) [17,18,19]. For some actinomycetes, overlaying the agar plates might lead to difficulties as the suspension washes away the mycelium, and due to the mixture, the producer is distributed over the agar plate. This often occurs when the producer is a fast-growing or sporulating strain. A careful platting of the indicator strain solution instead of overlaying the whole plate might solve this problem. Furthermore, an overlay with soft agar containing the indicator strain would simplify the procedure for producers that are stuck in the agar (e.g., *Streptomyces* strains) and are not washed away. For conducting the “direct” agar diffusion assay, suitable solid media should be applied. It is important that both strains (producer and test strain) can grow on the agar and the antibiotic production is ensured. The International Streptomyces Project-2 agar (ISP-2; was developed by Difco Laboratories for the International Streptomyces Project [43]) is often the first choice as it is a rich and clear medium. This increases the chances of antibiotic production and facilitates the visualization as well as the evaluation of the growth inhibition zones.

The ISP2 agar was also utilized for the modified variant of the “direct” agar diffusion assay with the purpose of adaptive laboratory evolution of actinomycetes [17]. According to the concept of this method, the potential antibiotic-producing strain is activated due to the competition against a target pathogen (trigger) in the serial transfer of the producer and exposure to the pathogen. It is expected that the competition increases the mutation rate. Those mutations which activate the biosynthesis of antibiotics or cause an increase in the production compared to the precursors strain are easily identified by the appearance of a halo and a larger zone of inhibition, respectively. With this system, a *Streptomyces clavuligerus* strain was evolved against the methicillin-resistant *Staphylococcus aureus* N315 [17]. In contrast to the unevolved *Streptomyces clavuligerus*, the evolved strain produced the compound holomycin, which inhibited the pathogen. 

In the agar plug-based diffusion assay, the producers (actinomycetes) are grown on a suitable solid medium and plugs are punched out of the agar using an agar punch device (often a sterile cork borer or a tip are used). The plugs are placed onto fresh plates containing the indicator strain. The plug-based diffusion assay has the advantage that the producer can be cultivated independently on different media. Thus, the testing of various antibiotic production conditions is feasible [21,22,23,24,27,44]. Recently, a 96-well microplate-based system, suitable for the screening of actinomycete strain collections in agar-plug assays, was established [20]. In this assay, agar plugs were generated by distributing agar in a modified microplate with a removable bottom and inoculating the solid medium with actinomycete spore suspensions. After incubation, the bottom was removed, and the agar plugs with the well-grown strains were pushed out of the wells using a special tool. This method allowed for the placing of actinomycete agar plugs onto test Petri dishes (plates containing the indicator strain) for agar-plug bioactivity assays. Even though “custom-made” equipment is required for conducting this assay, the procedure remains quite simple and enables the researchers to perform a medium-throughput screening. As the producer strains are cultivated independently of the indicator strain on separate agar plates, the users are more flexible and do not need to consider the compatibility of the medium for both strains, which is the case for the “direct” agar diffusion assay. 

The agar well diffusion method (agar “hole-based” diffusion assay) is similar to the agar plug-based diffusion assays.. While for the first method, the agar punch device is applied to obtain plugs with the producer, in the second approach, it is required for punching holes (diameter of 6 to 8 mm) in test agar plates [18,19,25,26,27,28,45,46]. These holes are filled with an antimicrobial solution (culture supernatant, extracts or purified antibiotic solution, volume of 20–100 mL) and the plates are incubated under suitable conditions depending upon the indicator strain. In contrast to the above-mentioned assays (“direct” and agar plug-based diffusion assay), the agar well diffusion method requires the preparation of a culture supernatant or the extract of the potential producer. On the one hand, this is unfavourable for compounds that cannot be extracted or are instable at the applied condition as the bioactivity might be missed or lost. On the other hand, these issues (loss of bioactivity, instability) could be defined as exclusion criteria to eliminate very challenging cases to save time and resources. In addition, this method is particularly convenient for the activity testing of fractions obtained during the chromatographic purification (e.g., preparative HPLC fractions) of a compound. 

Another option for the screening of culture supernatants [47], extracts [48], HPLC fractions [49] and purified antibiotics in solutions [49,50] is the agar disc diffusion. The procedure involves the preparation of test plates that are inoculated with an indicator strain and filter paper discs with the solution for an examination of the antibiotic activity. Typically, 10–100 µL are transferred onto a filter paper disc. For higher volumes, it is recommended to load the filter paper discs several times with a lower volume (e.g., 50 µL) and dry them in between to avoid overloading the disc and losing antibiotic solutions. In contrast to the “direct” agar diffusion and agar plug diffusion assay, the well- and agar disc diffusion method allows for a concentration-dependent examination of the material [51] and a rough determination of the minimal inhibitory concentration (MIC) [52] of pure compounds with a known concentration. Furthermore, based on the size of the inhibition zones for defined concentrations of a purified antibiotic substance, a standard calibration curve using the linear equation can be generated and applied to determine the concentration of such an agent in an unknown sample (semi-quantification) [53,54]. This concept is also utilized in liquid setups [55,56]. For example, microtiter plates are filled with medium containing the indicator strain and an antibiotic solution of a different concentration is added. Based on the measurements of the absorbance or optical density and the resulting calibration curve, the antibiotic concentration in undefined samples can be semi-quantified. Recently, such a system was established for *Streptomyces fradiae* to screen for tylosin “superproducers” [57]. 

Taken together, agar diffusion techniques are simple, fast and inexpensive methods for testing the antimicrobial activities of different material obtained and/or purified compounds. In case of non-purified material (e.g., culture supernatants, extracts), differentiating which compound is causing the inhibition of the indicator strain in an agar diffusion assay is not possible as some actinomycetes produce a mixture of several compounds that might result in a synergistic effect [7]. The quantification with agar diffusion assays is rather limited to purified agents or materials that are confirmed to contain only one compound that is active against the chosen indicator strain. Nevertheless, these assays are extremely useful for primary screenings, especially in cases where the compound is not characterized, and analytic methods do not yet exist. The accuracy and reproducibility of the agar diffusion methods is affected by factors such as agar nutrient content, thickness (volume) of the agar layer, uniformity of the agar, temperature, the interpretive criteria for the inhibition zones and breakpoints, and others [58]. Therefore, assays that are compared with each other or used for diagnostics must be conducted at defined conditions. Such standards for testing against bacteria and yeasts and their updates are, for example, published by the Clinical and Laboratory Standards Institute (CLSI) [59,60]. Finally, controls should be included to validate the procedure. Negative controls (solvent) eliminate the risk of misinterpretation when the antimicrobial activity is caused by the solvent (e.g., methanol, ethanol) that was, for example, used for resolving the concentrated extract after evaporation. As positive controls, known and confirmed antibiotics such as tobramycin [61,62] for Gram-negative indicator strains or erythromycin [63,64] for Gram-positive indicator strains can be applied. An inhibition zone for the positive control demonstrates that the assay is working and excludes potential technical issues. 

### 2.2. Thin-Layer Chromatography (TLC)–Bioautography

Thin-layer chromatography (TLC)–bioautography combines the separation and analysis technology of TLC with the detection of biological activity. The pioneers of the TLC–bioautography were Martin and co-workers [65] and Goodall, together with Levi [66]. In their experiments, paper chromatography (PC) was coupled with contact bioautography for the analysis of amino acids and penicillin, respectively. The term TLC was officially introduced by Fischer and Lautner [67]. Thereafter, TLC–bioautography was further developed and several types emerged [68] (e.g., agar diffusion (contact bioautography), direct bioautography and agar-overlay assay (immersion bioautography), high-performance thin-layer chromatography bioautography and D-TLC bioautography) which were recently described in another review [68]. Briefly, TLC involves the separation of components in a sample using a stationary (TLC plate) and mobile phase (organic solvent). The stationary phase is a thin adsorbent material layer (e.g., silica gel or aluminium oxide), coated onto an inert plate surface (usually glass, plastic or aluminium). Samples are spotted onto the TLC plate (the starting position is often marked on the plate) with a capillary spotter (e.g., five dips on every TLC plate with a 5µL microcap). It is recommended to spot the sample solution at one position of the TLC plate by transferring small amounts (spots) and drying the plate in between. This is an advantage for diluted samples as with this procedure, they become concentrated. At the same time, spotting too much material of very concentrated samples should be avoided because this reduces the quality of the separation. In addition, there should be enough space between the spots of different samples and the edges of the plate. After drying, the plate is placed vertically into a closed chamber with an organic solvent. The mobile phase migrates from the starting position towards the lid of the chamber (capillary forces). The mobile phase transfers the components into the same direction, however, since they have a differential affinity for the stationary and mobile phase, the components stop the migration at different positions on the TLC plate which results in various distances for each of the substances (spot on the TLC plate). As very similar substances have the same or almost the same chemical properties and affinity in this system, their spots often overlay and cannot be separated (mixture of compounds). The migration is monitored, and shortly before the solvent reaches the top of the plate, the plate is removed from the developing chamber. The solvent front is marked for the calculation of the retardation (retention factor (R_f_)) value and subsequently, the plate is dried. The plate should be evaluated under normal light, and in cases where the silica gel is impregnated with a fluorescent dye, it should also be evaluated under ultraviolet (UV) light (for green fluorescence: excitation at λ = 254 nm; for blue fluorescence excitation at λ = 365 nm). Depending on the chemical properties of the compound, different, commercially available visualization reagents can be applied [69,70,71]. The R_f_ value is the ratio of distance travelled by the compound (spot on the plate) to that of the solvent front:

$$R_{f}=\frac{{Z_{a}}^{\;(\mathrm{migration}\;\mathrm{distance}\;\mathrm{of}\;\mathrm{the}\;\mathrm{compound})}}{Z_{b\;(\mathrm{migration}\;\mathrm{distance}\;\mathrm{of}\;\mathrm{solvent}\;\mathrm{front})}}$$

For linear development, Z_a_ is the distance migrated by the compound from its origin (starting position of the separation) to the position of the spot on the plate, while Z_b_ is the distance migrated by the mobile phase (from the starting position to the solvent front).

The second step of TLC–bioautography is the in situ biological activity detection. This can be achieved by the transfer of the compound to an agar test plate that is inoculated with an indicator strain (agar diffusion also called contact bioautography). Therefore, the TLC with the side containing the compounds separated in the thin adsorbent material layer is laid on the test agar plate, and after the few minutes or hours removed, the single spots are scraped off the layer (powder is transferred onto the test agar plate). If the tested compound is active against the indicator strain, an inhibition zone will be visible after the required incubation time [33,34,35].

Furthermore, another and faster detection of the activity is direct bioautography. Here, the TLC plate with potential bioactive agents is dipped into or sprayed with a suspension containing the indicator strain and incubated at conditions allowing the growth of the indicator strain [72]. For the evaluation of antimicrobial activity, tetrazolium salts are sprayed onto the TLC plate. As living cells produce dehydrogenases, which convert the tetrazolium salts to their intensely coloured formazan products, the areas where the strain is growing change colour. In contrast, parts of the TLC that contain an antibacterial compound remain clear and can be easily distinguished from the background [36,37]. 

The agar overlay bioassay is a combination of the agar diffusion and direct bioautography. After separation on the TLC plate, the plate is overlaid with inoculated agar medium. It is important to cool down the agar (approximately 55 °C) to avoid killing the indicator strain. During the incubation at suitable conditions (depending on the indicator strain), the compounds diffuse, and their activity is manifested by the appearance of the inhibition zone. Similar to direct bioautography, the indicator strain is stained with a tetrazolium dye enabling the evaluation of its growth and identification of inhibition zones caused by active compounds. 

TLC–bioautography has been mainly used for the detection of antibacterial and antifungal activity [38,39,73,74]. For example, Grzelak et al. [35] applied all three technics (agar diffusion (contact bioautography), direct bioautography and agar-overlay assay (immersion bioautography) to test several actinomycete-derived compounds, showing a wide range of polarities for antitubercular (anti-TB) activity [35]. 

In addition to agar diffusion methods, TLC–bioautography is a fast, simple, sensitive, and reliable approach which does not require complicated equipment for screening antibiotics and other compounds. The separation on the TLC plate facilitates the analysis of the single spots (compounds). However, similar substances might result in a spot containing a mixture. Thus, for further characterization or dereplication purposes, it is useful to combine the TLC with a high-performance liquid chromatography (HPLC), liquid chromatography mass spectrometry (LC-MS) and/or other methods. This can be carried out independently [39] (samples are analysed in parallel using TLC–bioautography and HPLC/MS methods) or the spots (compounds) obtained from the TLC are further characterized (e.g., spots are scraped from the plate, extracted and the extracts are analysed by HPLC/MS or other methods) [38,40,41,42]. Details on chromatographic methods can be found in comprehensive reviews [75,76,77,78,79]. 

## 3. Secondary Screening: Target-Based Assays 

**Figure 1:**
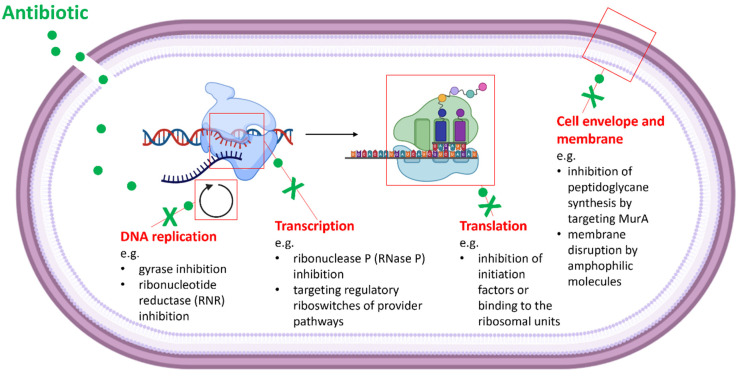
Overview on common antibiotic targets and the inhibition of the essential cellular processes. (The figure was drawn using BioRender.com (2022). Retrieved from https://app.biorender.com/biorender-templates) (accessed on 13 September 2022).

Target identification is one of the most crucial steps in drug development. In the past, various strategies for the characterization of drug targets were developed [80,81,82,83]. These include genomic approaches, phenotypic profiling/screening and biochemical strategies. Computational approaches involve genome analysis and the identification of potential resistance genes. Therefore, natural antibiotic producers as well as other naturally resistant strains (e.g., resistant pathogens) and drug-resistant clones—that were obtained after the exposure of the originally susceptible strain to the antimicrobial agent—are used. As the genomes might encode new; so far, unexplored; or multiple resistance factors, this method is often not sufficient for the unambiguous identification of the antibiotic target and elucidation of its MOA. Typically, the in silico analysis is followed by more specific whole cell assays that apply recombinant indicator strains (e.g., reporter systems, an overexpression mutant library for essential genes such as the ASKA library [84,85]), diverse biochemical screenings (e.g., affinity methods (pull-down assays [86]) and in vitro assays with potential targets). The screenings often use cell wall compartments, DNA, RNA, ribosomes and enzymes of metabolic pathways as macromolecular targets (Figure 1). The second part of this review will lead the reader through such in vivo whole cell screenings and in vitro target-based assays (Table 2). Some of them were established to high throughput methods, and others will follow.

### 3.1. Assays for Targeting Cell Wall 

The cell envelope is a complex multi-layered structure that protects the cell, shapes the cell, provides stability and rigidity and plays a central role in the communication with the environment (e.g., sensing and transport of nutrients) [116,117,118,119]. In addition, the cell wall components are essential for many other processes in the cell (e.g., growth, cell division, cell wall recycling) [120,121,122,123]. Consequently, finding antibiotics that target the cell wall of pathogens is one of the major goals in the antibiotic discovery field [124,125,126]. As the cell wall of Gram-positive and Gram-negative bacteria differs in its composition [96,127,128] (e.g., in contrast to Gram-positive bacteria, Gram-negative strains possess an outer membrane (OM)), it is possible to differentiate in the inhibition by using antibiotics that specifically target only one of the two groups of bacteria. Indeed, the cell wall became a very popular target, and thus, several assays for the screening of compounds that act as cell wall inhibitors have been developed to combat bacterial infection. 

One example is a whole-cell assay to test agents, which interferes with peptidoglycan (PG) biosynthesis, including the inhibition of cell wall recycling [95]. In this assay, ^14^C-labeled UDP-N-acetylglucosamine (UDP-GlcNAc) was fed to pre-treated *E. coli* ATCC 47076 cells (subjected to freezing and thawing). The utilization of ^14^C-labeled UDP-GlcNAc facilitated the direct detection of cross-linked PG which indicated a PG recycling and regeneration of the cell. When inhibitors (antibiotics) of these processes were added to the samples, the optical density (OD_600_) was affected, and lower radioactivity was detected in comparison to the negative compound (without addition of the antibiotic). The functionality of the assay was confirmed using known PG inhibitors (e.g., fosfomycin, bacitracin, flavomycin) which inhibited the formation of radiolabelled PG and compounds that do not target the enzymes of the PG pathway as a negative control (no effect was observed for kanamycin/streptomycin, norfloxacin). Applying the whole-cell approach for peptidoglycan biosynthesis inhibitors in combination with an enzymatic assay with purified enzymes resulted in the identification of two new compounds (Cpd1 and Cpd2), which specifically block enzymes of PG synthesis (e.g., MurA) [95]. At the concentration of 50 μM Cpd1 and Cpd2, the agents inhibited the assay by 25 and 50%, respectively.

The advantage of this assay is the relatively easy implementation. However, it usually requires expensive radioactively labelled substrates, the respective facility and trained staff to work with radioactivity. This might limit the high-throughput screening (HTS).

An impressive method for the visual and spectroscopic detection of bacteria (in particular, bacterial contaminations) was developed by Silbert, et al. [115]. The principle involves an interaction of membrane-active compounds secreted by bacteria with agar-embedded nanoparticles. The nanoparticles comprise phospholipids and the chromatic polymer polydiacetylene (PDA) to simulate a membrane. It has been demonstrated that molecules which are produced by bacteria affect the PAD, leading to blue-to-red transformations with an intense fluorescence emission [115]. This can be measured by conventional HTS instruments. The spectroscopic detection method was implemented for the screening of actinomycete-derived extracts after an activity was detected in a primary screening using agar diffusion assays [129]. Therefore, the extracts which were resolved in DMSO were incubated for 1 h with phospholipid/PDA (vesicle solution used as a model for a membrane). In cases of extracts which contained molecules interacting with the artificial membrane and/or disruption the vesicles, the colour was changed, and the fluorescence emission was detected by a UV-Vis spectrophotometer. For two actinomycete-derived extracts, the blue-to-red transformations were detected. This indicated that these samples harbour compounds which target the bacterial membrane. 

The assay is a very convenient method, as colorimetric responses can be measured, thus, facilitating the quantification. Problems might arise if the extracts contain coloured substances, as this might interfere with the detection method. In such a case, additional steps (e.g., TLC–bioautography, preparative HPLC) are recommended to separate the compounds and re-test them one-by-one.

Recently, an interesting assay was developed whereby not the metabolites produced by actinomycetes were screened, but the actinomycete itself was exploited for developing a screening method [87]. Gosschalk and co-authors focused on finding the inhibitors of sortase enzymes which are attractive drug targets. These enzymes attach virulence factors to the surface of *Staphylococcus aureus* and other relevant bacterial pathogens. Blocking the sortase enzymes would lead to the loss of the virulence factors. To develop an effective screening assay, *Actinomyces oris* was applied. This strain exhibits sortase-dependent growth in laboratory conditions and thus, it is particularly suitable as a “sensor” for sortase-inhibitors. To eliminate small molecules that impaired *A. oris* growth via processes unrelated to sortase, mutants of *A. oris* were generated and a secondary screening coupled with a primary screening (the wild type of *A. oris* was used in the primary screening) was introduced. Based on this phenotype, a HTS was established. This delivered two candidates, whereby sortase-inhibitory activity was also confirmed in vitro [87].

Although a primary and secondary screening is required for the specific identification of sortase inhibitors, this system is a simple and relatively inexpensive opportunity.

### 3.2. Assays for Inhibitors of DNA Synthesis

Every future generation of cells must be equipped in a newly synthesized chromosome. Thus, the inhibition of DNA synthesis prevents cell propagation. The most prominent target for the inhibition of these processes in bacteria is DNA gyrases (topoisomerases) as they have multiple roles in DNA replication, recombination, and transcription [130,131,132,133]. It has been shown that many quinolone antibiotics originally isolated from actinomycetes (such as nalidixic acid [134]) act as potent DNA gyrase inhibitors [132,135]. Assays for screening DNA synthesis inhibitors include cell-based high-throughput bioluminescence screens [100,101]. For example, Moir, et al. [101] fused a luciferase operon to a promoter that responds to DNA damage caused by reduced gyrase levels in *Pseudomonas aeruginosa* (*P. aeruginosa* with chromosomal inserted *luxCDABE* luciferase genes). The promoter (*PA0614*) was derived from the pyocin gene-encoding region. Pyocins are toxic bacteriocins, produced by *P. aerogionsa*, that kill closely related *Pseudomonas* strains. *PA0614* responded to ciprofloxacin and decreased GyrA levels. Consequently, in case a compound that interacts with the promoter is added to the assay, the gyrase expression is repressed. At the same time, the expression of the *lux* genes is upregulated (luminescence that can be detected by a luminometer). The generation of the recombinant strain resulted in coupling the transcriptional regulatory response produced by the depletion of an antibacterial target (gyrase) to a suitable reporter. This reporter assay was used for the screening of 2000 known compounds. The screening revealed that 13 of them were confirmed gyrase inhibitors, 10 out of the 13 inhibitors were quinolones, but the remaining 3 were non-quinolone structures (mechlorethamine-, furazolidone-, and nitrofuran-like structures).

This whole-cell bioluminescent assay enables the researchers to specifically screen for gyrase inhibitors in high throughput. The generation of the recombinant strain means that additional cloning and genetic manipulation steps must be included unless the system can be obtained from other labs. 

In addition to the whole cell assays, enzymatic in vitro approaches can be applied for screening compounds that target the enzymes involved in DNA synthesis. PCR-based enzymatic assays are particularly useful. Tholander et al. presented a PCR-based assay for ribonucleotide reductase (RNR) activity measurements in a microplate format [104]. RNR catalyses the reduction in the four ribonucleotides necessary for DNA synthesis to deoxyribonucleotides, and thus, is a rate-limiting enzyme of DNA synthesis [136,137,138]. Although the RNR is a frequent target for antibiotics, possible inhibitors are not well studied due to the laborious experimental procedures. PCR-based assays allow for the quantification of the reduction in ribonucleoside-5-diphosphates (NDPs) to deoxynucleoside diphosphates (dNDP) that are catalysed by RNR. Therefore, SYBR green dye (asymmetrical cyanine dye used for staining nucleic acids) is added to the sample, and after binding to the DNA, fluorescence can be detected. The higher the RNR activity, the more the product is generated in the PCR, and the stronger the fluorescence signal is. Using this assay, 1364 compounds were tested for the inhibition of class 1 RNR of *P. aeruginosa*. Within these substances, 110 have shown a 50% inhibition of RNR activity" (enzyme activity) is correct, and 27 of them revealed an inhibition of over 90%, with IC_50_ values ranging from 30µM to 200µM. These 27 compounds were further tested for dose-dependent responses and for their impact on *P. aeruginosa* growth and proliferation. Four of them have shown effects on *P. aeruginosa* that were comparable to those of tetracycline and carbenicillin. One of these four potent inhibitors was streptonigrin (from *Streptomyces flocculus*) [104].

The two examples of assays presented in this section enable the screening for RNR inhibitors. The first (cell-based high-throughput bioluminescence) has the advantage that the inhibitors must pass the cell envelope to reach the target, and hence, the screen sorts out all those candidates which cannot pass this barrier. The second is probably more specific, but it requires the purification of the RNRs for testing. In this case, it would be interesting to examine if a crude extract containing the protein could be applied to simplify the procedure and reduce the costs for the purification of the enzyme.

### 3.3. Assays for the Inhibitors of Transcription and Translation

Transcription (RNA synthesis) and translation (protein synthesis) are indispensable processes in every living cell. Therefore, the inhibition or complete abolishment of the transcription or translation in pathogenic bacteria is a desired strategy to fight pathogens causing severe infections. Furthermore, the molecular mechanisms of the transcription and translation in prokaryotes and eukaryotes differ from each other (e.g., the subunits of the DNA-dependent RNA polymerases of bacteria and eukaryotes are fundamentally different). This increases the chances of a specific inhibition of the transcription/translation in bacteria without disrupting these essential processes in eukaryotic cells, which is an extremely important criterium for drug development. 

To screen for new transcription/translation inhibitors, diverse assays were developed [139,140,141,142,143,144,145]. Those include biosensor assays (I) (real-time measurement of protein inhibition using luciferase assays [146], stress response assays [147], antibiotic detection assays [148,149,150], attenuation-based dual fluorescent reporter assays [151], panel of reporter strains that lack antibiotic resistance [97], transcriptional sensors based on promoter libraries [152,153] etc.); in vitro methods (II) (in vitro protein synthesis inhibition assays [103,154,155], toe-printing of antibiotic-stalled ribosomes [105], SPARK-sensitive method for monitoring peptidyl transferase activity [156], antibiotic binding to a fluorescently labelled ribosome [157] etc.); in vivo methods (III) (fluorescent microscopy and bacterial cytological profiling [158], proteomics-based methods [159,160], resistance-based assays to sensor the mechanism of action [105] etc.). A comprehensive overview on these technics was provided by Osterman and co-authors [161]. Here, we focus on three examples of assays in which transcription/translation inhibitors, isolated from actinomycetes, were tested or identified. 

In vitro transcription/translation assays (also referred to as cell-free protein synthesis systems) are powerful tools that are used in basic research for answering different scientific questions, such as finding the target of a drug, as well as in drug discovery for screening. To conduct the assay, all components for transcription and translation must be present in the sample mixture. Cell extracts [162,163,164,165] (e.g., from *E. coli*) are often applied instead of purified enzymes. Further components, such as nucleotides and amino acids as substrates for transcription and translation, fructose-1,6-bisphosphate for energy supplies and a reporter system (e.g., pET28-egfp), are added to the sample to ensure the transcription/translation of the reporter gene/protein. In samples which were supplemented with an inhibitor of the transcription or translation, there is either no signal or a weaker signal (e.g., fluorescence) compared to the control. As this correlates with the production of the reporter protein (e.g., eGFP), the inhibition of transcription/translation can be detected by the respective spectrometry method (e.g., using fluorometer). Such a transcription/translation assay was applied, for example, to investigate the activity of kirromycin derivatives produced by an engineered mutant of *Streptomyces collinus Tü* 365 [103]. The target of kirromycin (elongation factor Tu (EF-Tu)) was previously identified [166,167]. In case the in vitro transcription/translation assay is applied to compounds which lead to an inhibitory effect, but where the target is not known, further characterization might be necessary. This can be achieved by specific in vitro methods that “present” the respective component (target) for interaction. An example thereof is an assay (the real-time fluorescence polarization activity assay (FP/FA assay)) in which the bacterial ribonuclease P (RNase P) was used as a target and was exposed to diverse inhibitors [168]. The RNase P (endonuclease) catalysed the cleavage of the 5′ leader sequence from precursor tRNAs (pre-tRNAs), resulting in the generation of the mature tRNA with a 5’ end. The method allows for the detection of compounds that bind to the pre-tRNAs and those which inhibit the RNase P. The assay was validated with antibiotics from actinomycetes, neomycin B and kanamycin B, and optimized for HTS. A library harbouring 2880 compounds was screened. Iriginol hexaacetate was identified as a new inhibitor of *Bacillus subtilis* RNase P [168]. 

Another interesting approach is the use of bacterial riboswitches for HTS methods of antibacterial drug candidates [169,170,171]. Riboswitches are RNA elements which can bind to metabolites and regulate gene expressions, mainly in bacteria [172]. Since the discovery of the first riboswitches, which were described as RNA-based intracellular sensors of vitamin derivatives [173,174], many new riboswitches have been identified, characterized and assigned to 28 experimentally validated classes. As natural or synthetic ligand analogues (small molecules) can bind the riboswitches and stop their regulatory functions, they represent a promising target for antibiotics [175]. To screen for antibiotics that bind to bacterial riboswitches, assays that utilize reporter-based systems have been developed [169,176,177]. For example, Lee et al., used the *lacZ* reporter system in *B. subtilis* and demonstrated that roseoflavin (naturally produced by *Streptomyces davawensis*), a chemical analog of flavin mononucleotide FMN and riboflavin [178,179,180], binds to the FMN riboswitch and downregulates the expression of the FMN riboswitch-*lacZ* reporter gene [177]. FMN riboswitch regulates the expression of genes which are involved in the biosynthesis and transport of riboflavin (vitamin B2). Riboflavin is a precursor of the essential FMN and flavin adenine dinucleotide (FAD). The binding of roseoflavin to the riboswitch leads to the repression of the riboflavin biosynthesis and transport. Consequently, bacteria that respond to roseoflavin (or other specific agents) are inhibited (antibiotic effect). A limitation for establishing these assays—which occurs not only in case of roseoflavin, but has also been observed many times for antibiotics—is the emergence of resistance [181,182,183,184,185]. For example, mutations in the region coding for the FMN riboswitch, which confer the resistance to roseoflavin, were found in *Listeria monocytogenes* [175,186]. Therefore, the identification of suitable riboswitches and the development of HTS methods that facilitate the discovery of compounds which specifically interact with the riboswitches should be considered in the field. 

### 3.4. Assays for Identification of Essential Enzymes Inhibitors 

In the development of specific assays for the identification of potential antibiotics or their targets, enzymes catalysing essential processes in the bacterial cell are often used. Ideally, these enzymes should be absent or fundamentally different in eukaryotic cells to avoid toxicity. Usually, the activity and specificity for the bacterial target is tested first, followed by toxicity screens with eukaryotic cell lines. 

Many of the enzyme-based assays utilize classical targets such as enzymes that are involved in the synthesis and/or recycling of cell wall compartments, especially peptidoglycan (see section “Assays for targeting cell envelope”, MurA). Other examples include the gyrase (essential for DNA replication), ribonucleotide reductase (RNR) (essential for DNA synthesis, see section “Assays for inhibitors of DNA synthesis”), bacterial ribonuclease P (required for RNA synthesis) as well as various enzymes catalysing important steps of the vitamin-, amino acid- or coenzyme-biosynthesis [107,108,110,175]. Finally, proteins of the ribosomal subunits as well as assembled ribosomes are exposed to the tested compounds in such assays (see section “Assays for inhibitors of transcription and translation”) [161]. Some of these methods involve a pulse and chase labelling procedure to measure the kinetics of the ribosomal subunit formation or procedures facilitating the examination of ribosome reformation after antibiotic removal for studying post-antibiotic effects [187,188]. Indeed, a significant number of the antibiotics isolated from actinomycetes target the bacterial ribosome, thus underlining the importance of methods enabling the screening and identification [145,189,190,191,192,193] of the antimicrobial agents.

In addition, histidine kinases (HK) were included as a target in the search for antibacterials [194,195,196]. HK are membrane receptors, which control a variety of cellular responses (e.g., virulence, secretion systems and antibiotic resistance). They function in two-component signal transduction pathways. Two-component systems (TCSs) consist of a HK and a response regulator (RR). The inhibition of the TCS might kill the host or reduce the resistance of bacteria to antibiotics by enhancing stress responses, such as the cell wall stress response. For example, a methicillin-resistant *Staphylococcus aureus* strain became susceptible after inhibition of the TCS [197]. Thus, the establishment of efficient screening assays for inhibitors of the TCS, including HK, is promoted in the antibiotic drug discovery. For instance, the recently published immuno-dot blot assay is a promising technic for the detection of HK activity and their profiling [198]. In vitro kinase assays such as autophosphorylation approaches with γ-32P-ATP [199,200] are often used where the phosphorylated protein (histidine phosphorylation in the histidine kinase) is quantitated by phosphorimaging. In screenings, active inhibitors reduce or prevent the phosphorylation (either no signal or a weaker signal compared to the positive control). For details on further approaches and recent advances in targeting histidine kinases, the reader is redirected to other reviews and research papers [194,196,201,202,203,204,205,206].

## 4. Summary and Conclusions

The emergence of multi-to-pan drug-resistant pathogens and their global spread, and the fact that big pharma has shut down antibiotic research and development because of the lack of financial incentives, are the main reasons that unleashed the antibiotic crisis. The World Health Organisation (WHO) estimated that antimicrobial resistance might lead to 10 million deaths a year by 2050 [207]. Thus, finding and developing new antibiotics to overcome or at least attenuate the consequences of the antibiotic crisis are of global interest. However, research and development require the financial support of governments and funding agencies as well as close collaborations between the industry and academia. To accelerate progress regarding antibiotic discoveries, methods facilitating primary and secondary screenings of crude extracts and/or purified compounds are indispensable. These assays are often implemented for the screening of actinomycete products, as these strains are a confirmed source of very potent antibiotics. Certainly, the screening methods are applied and can be further optimized for the characterization of antimicrobial activities in material obtained from other producers or sources (e.g., chemically synthesized compounds). Primary screening assays (e.g., agar diffusion assays, TLC-based methods, whole cell assays) offer simple, fast and inexpensive opportunities for checking the antibiotic activity in a sample (e.g., culture supernatant, extract). This is particularly demanded when new potential producers such as actinomycetes are isolated and examined for their bioactive products. Once the isolate’s product shows an inhibition of bacterial test strains and the results of dereplication (e.g., using HPLC, HPLC-MS, high-performance liquid chromatography–high resolution mass spectrometry (HPLC-HRMS) and chemical databases) strongly indicate a new entity, the compound is subjected to further characterization. This often requires purification, as crude extracts are mixtures of compounds which interfere with many downstream steps in structure elucidation and target identification.

In secondary screening, mostly target-based assays are applied—these allow for the exploration the mode of action. In the past, traditional targets, such as cell wall compartments, DNA, RNA, ribosomes, metabolic enzymes and other proteins, were utilized in screening assays [208,209,210,211]. In contrast to the primary screening assays, the secondary screens usually involve additional molecular steps (e.g., cloning, generation of mutants, protein purification) and the respective equipment for conducting the measurements. Although the secondary screening seems to be technically more challenging, it often offers opportunities for HTS. Moreover, the outcome delivers valuable knowledge for understanding the MOA of antibiotic drugs, which is difficult to obtain with unspecific primary screening methods. 

As there are still compounds with uncharacterized MOA [211] or antibiotics where the direct physical interactions with the target (e.g., ribosome) are largely unexplored [145] (e.g., AZ7), the optimization and development of new assays, including novel targets that can be used as tools in antibiotic development and approval, are essential [212,213,214]. Furthermore, it is expected that new compounds with unknown MOA will be isolated from natural sources in the future. Therefore, establishing platforms that enable primary and secondary screenings as well as combining assays for testing different targets [100,152] at once will speed up the characterization of the antibiotic activities. 

## Figures and Tables

**Table 1:** Examples of diffusion- and bioautography-based assays for the detection of antimicrobial activity.

Method	Specificity	Robustness	Difficulty	Estimated Time (Experiment-Result)	Costs	Comments	References
Agar Diffusion Assays							
“Direct” agar diffusion assay	+ *	+	(+) Easy	5–15 days (depends on the producer and indicator strain)	(+)	- Basic equipment is sufficient. - Media must be compatible with the producer and indicator strain.	[17,18,19]
Agar plug diffusion assay	+ *	++	(+) Easy	5–15 days (depends on the producer and indicator strain)	(+)	- Basic equipment is sufficient. - Does not require media compatibility for the producer and indicator strain (flexible).	[20,21,22,23,24]
Agar well diffusion assay	+ *	++	(+) Easy	12–24 h (excluding the preparation of the material (e.g., extract) for testing; depends on the indicator strain)	(+)	- Basic equipment is sufficient. - Suitable for the screening of culture supernatants, extracts, purified compounds, etc.	[19,25,26,27,28]
Agar disc diffusion assay	+ *	++	(+) Easy	12–24 h (excluding the preparation of the material (e.g., extract) for testing; depends on the indicator strain)	(+)	- Basic equipment is sufficient. - Suitable for the screening of culture supernatants, extracts, purified compounds, etc. - Standardized assays enable a semi-quantification in case of known antibiotics.	[29,30,31,32]
Bioautography assays							
Thin-layer chromatography (TLC)–bioautography	+(+) **	++	+	12–24 h (excluding the preparation of the material (e.g., extract) for testing; depends on the indicator strain)	+	- Basic equipment is sufficient. - TLC plates are commercially available. - Suitable for the screening of extracts, fractions from purification and purified compounds.	[33,34,35,36,37]
Combined methods (e.g., TLC–bioautography and HPLC/LC-MS ***)	++	++	+	12–24 h (excluding the preparation of the material (e.g., extract) for testing; depends on the indicator strain)	+++	- HPLC, LC-MS instruments are required. - Reference compounds and libraries enable quantitative and qualitative measurements.- Better outcomes (assignment of the bioactivity to a peak (compound) is possible).	[38,39,40,41,42]

* Differentiation between activity against Gram-positive and Gram-negative bacteria. ** Bioactivity can be assigned to spots (compounds) on the TLC. *** HPLC: High-performance liquid chromatography, LC-MS: liquid chromatography mass spectrometry.

**Table 2:** Examples of target-based assays for the detection of antimicrobial activity.

Method	Description	Specificity	Robustness	Difficulty	Estimated Time (Experiment-Result)	Costs (Considering Equipment)	References
In Vivo (Whole-Cell) Assays							
Cell viability/cytotoxicity assays	- Detection of living or dead cells by measuring the absorbance (or using colorimetry). - Suitable for killing curves (testing of antibiotic effects) and MIC * determination. - HTS ** is possible.	++	+++	+	6–24 h (excluding the preparation of the material (e.g., extract, purified compound; depends on the test strain)).	++	[57,87,88,89,90,91,92,93]
Whole-cell assays using isotope-labelling and radioactivity	- Introduction (e.g., feeding) of isotope-labelled substrates and detection of radioactivity. - Suitable for the identification of antibiotic targets (e.g., peptidoglycan, histidine kinases). - HTS ** is possible but unsustainable.	+++	++	+++	12–42 h (excluding the preparation of the material (e.g., extract, purified compound; depends on the test strain).	+++	[94,95,96]
Whole-cell assays using reporter systems and bioluminescence or fluorescence	- Introduction of reporter systems (e.g., *lux, egfp*) or organic fluorescent probes and detection of bioluminescence or fluorescence. - Often requires the genetic manipulation of the test strain. - Suitable for the identification of antibiotic targets (e.g., gyrase, ribosome, riboswitches). - HTS ** is possible.	++(+)(e.g., autofluorescence (background) might influence the measurements).	+++	++(+)	12–42 h (excluding the generation of mutants and preparation of the material (e.g., extract, purified compound; depends on the test strain).	++	[97,98,99,100,101]
In vitro assays							
In vitro transcription/translation assays	- Use of transcription/translation components (e.g., cell-free extracts or purified enzymes) in an in vitro reaction. - Often reporter systems (e.g., *egfp* encoding plasmids) are applied, which facilitate the detection of the protein (e.g., *egfp*). - Suitable for the identification of antibiotic targets (e.g., ribosome, translation and transcription factors). - HTS ** is possible.	+++	++(+)	++(+)	2–6 h (excluding the preparation of the cell-free extract and the material (e.g., extract, purified compound).	++(+)	[102,103]
Enzymatic assays (using purified enzymes)	- Use of purified enzymes in an in vitro reaction. - Often, labelled substrates are used (detection of bioluminescence, fluorescence etc.). - Suitable for the identification of antibiotic targets (e.g., histidine kinases, ribonuclease P, cell wall synthesizing enzymes, metabolic enzymes etc.). - HTS ** is possible.	+++	++(+)(Strongly depends on the stability of the enzyme and substrates).	+++	2–24 h (excluding the protein purification and preparation the material (e.g., extract, purified compound).	++(+)	[87,95,104,105,106,107,108,109,110]
Vesicle-based methods	- Use of phospholipid vesicles (membrane model). - Suitable for drug delivery and the investigation of antibiotic–membrane interactions - Often nanotechnology is involved.	+++	+(+)	+++	Strongly depends on the used system.	++(+)	[111,112,113,114,115]

## Data Availability

Data sharing not applicable.

## Abbreviations

AMR	antimicrobial resistance
BGC	biosynthetic gene clusters
dNDP	deoxynucleoside diphosphates
EF-Tu	elongation factor Tu
FMN	flavin mononucleotide
FAD	flavin adenine dinucleotide
FP/FA	real-time fluorescence polarization activity assay
HK	histidine kinases
HPLC	high-performance liquid chromatography
HTS	high-throughput screening
IC	inhibitory concentration
LC-MS	liquid chromatography mass spectrometry
MOA	mechanism of action
NDP	ribonucleoside-5-diphosphate
OD	optical density
OM	outer membrane
PG	peptidoglycan
Pfams	protein family domains
PDA	polydiacetylene
Rf	retention factor
RNR	ribonucleotide reductase
RNase P	ribonuclease P
TLC	thin-layer chromatography
UV	ultraviolet
UDP-N-acetylglucosamine	uridine-diphosphate-N-acetylglucosamine

## References

[1] World Health Organization (WHO) (2021). Antimicrobial Resistance. https://www.who.int/news-room/fact-sheets/detail/antimicrobial-resistance.

[2] Genilloud O. (2017). Actinomycetes: Still a source of novel antibiotics. Nat. Prod. Rep..

[3] Ziemert N., Alanjary M., Weber T. (2016). The evolution of genome mining in microbes–a review. Nat. Prod. Rep..

[4] Medema M.H., Fischbach M.A. (2015). Computational approaches to natural product discovery. Nat. Chem. Biol..

[5] Li M.H., Ung P.M., Zajkowski J., Garneau-Tsodikova S., Sherman D.H. (2009). Automated genome mining for natural products. BMC Bioinform..

[6] Kalkreuter E., Pan G., Cepeda A.J., Shen B. (2020). Targeting bacterial genomes for natural product discovery. Trends Pharmacol. Sci..

[7] Challis G.L., Hopwood D.A. (2003). Synergy and contingency as driving forces for the evolution of multiple secondary metabolite production by *Streptomyces* species. Proc. Natl. Acad. Sci. USA.

[8] Malit J.J.L., Leung H.Y.C., Qian P.-Y. (2022). Targeted Large-Scale Genome Mining and Candidate Prioritization for Natural Product Discovery. Mar. Drugs.

[9] Hug J.J., Bader C.D., Remškar M., Cirnski K., Müller R. (2018). Concepts and methods to access novel antibiotics from actinomycetes. Antibiotics.

[10] Zhang Q.-W., Lin L.-G., Ye W.-C. (2018). Techniques for extraction and isolation of natural products: A comprehensive review. Chin. Med..

[11] Abdelmohsen U.R., Sayed A.M., Elmaidomy A.H. (2022). Specialty Grand Challenge: Natural Products Extraction and Isolation-Between Conventional and Modern Techniques. Front. Nat. Prod..

[12] Pauli G.F., Chen S.-N., Friesen J.B., McAlpine J.B., Jaki B.U. (2012). Analysis and purification of bioactive natural products: The AnaPurNa study. J. Nat. Prod..

[13] Atanasov A.G., Zotchev S.B., Dirsch V.M., Orhan I.E., Banach M., Rollinger J.M., Barreca D., Weckwerth W., Bauer R., Bayer E.A. (2021). Natural products in drug discovery: Advances and opportunities. Nat. Rev. Drug Discov..

[14] Fleming A. (1929). On the antibacterial action of cultures of a penicillium with special reference to their use in the isolation of *B. influenzae*. Br. J. Exp. Pathol..

[15] Amsterdam D., Balows A., Hausler W.J., Ohashi M., Turano A., Lennete E.H. (1988). Principles of Antibiotic Testing in the Laboratory. Laboratory Diagnosis of Infectious Diseases: Principles and Practice.

[16] Heatley N. (1944). A method for the assay of penicillin. Biochem. J..

[17] Charusanti P., Fong N.L., Nagarajan H., Pereira A.R., Li H.J., Abate E.A., Su Y., Gerwick W.H., Palsson B.O. (2012). Exploiting adaptive laboratory evolution of *Streptomyces clavuligerus* for antibiotic discovery and overproduction. PLoS ONE.

[18] Singh V., Haque S., Singh H., Verma J., Vibha K., Singh R., Jawed A., Tripathi C. (2016). Isolation, screening, and identification of novel isolates of actinomycetes from India for antimicrobial applications. Front. Microbiol..

[19] Das R., Romi W., Das R., Sharma H.K., Thakur D. (2018). Antimicrobial potentiality of actinobacteria isolated from two microbiologically unexplored forest ecosystems of Northeast India. BMC Microbiol..

[20] Ortlieb N., Klenk E., Kulik A., Niedermeyer T.H.J. (2021). Development of an agar-plug cultivation system for bioactivity assays of actinomycete strain collections. PLoS ONE.

[21] Mazza G. (1983). Rapid assay for detection of microorganisms producing DNA-damaging metabolites. Appl. Environ. Microbiol..

[22] Jiménez-Esquilín A., Roane T. (2005). Antifungal activities of actinomycete strains associated with high-altitude sagebrush rhizosphere. J. Ind. Microbiol. Biotechnol..

[23] Elleuch L., Shaaban M., Smaoui S., Mellouli L., Karray-Rebai I., Fourati-Ben Fguira L., Shaaban K.A., Laatsch H. (2010). Bioactive secondary metabolites from a new terrestrial *Streptomyces sp.* TN262. Appl. Biochem. Biotechnol..

[24] Sarmiento-Vizcaíno A., Martín J., Reyes F., García L.A., Blanco G. (2021). Bioactive Natural Products in Actinobacteria Isolated in Rainwater From Storm Clouds Transported by Western Winds in Spain. Front. Microbiol..

[25] Kondo S. (1976). Punch Hole Method. A Simplified Bio-Assay Technique of Antibiotic Concentrations. Laboratory Aspects of Infections.

[26] Horváth G., Bencsik T., Ács K., Kocsis B. (2016). Sensitivity of ESBL-producing gram-negative bacteria to essential oils, plant extracts, and their isolated compounds. Acad. Press Amst..

[27] Saleem H.G.M., Aftab U., Sajid I., Abbas Z., Sabri A.N. (2015). Effect of crude extracts of selected actinomycetes on biofilm formation of *A. schindleri*, *M. aci*, and *B. cereus*. J. Basic Microbiol..

[28] Souza M.J., Bittencourt C.F., da Se Souza Filho P. (2004). Microbiological assay for enrofloxacin injection. Int. J. Pharm..

[29] Lu Q.-P., Ye J.-J., Huang Y.-M., Liu D., Liu L.-F., Dong K., Razumova E.A., Osterman I.A., Sergiev P.V., Dontsova O.A. (2019). Exploitation of potentially new antibiotics from mangrove actinobacteria in maowei sea by combination of multiple discovery strategies. Antibiotics.

[30] Alam A., Tanveer F., Khalil A.T., Zohra T., Khamlich S., Alam M.M., Salman M., Ali M., Ikram A., Shinwari Z.K. (2021). Silver nanoparticles biosynthesized from secondary metabolite producing marine actinobacteria and evaluation of their biomedical potential. Antonie Van Leeuwenhoek.

[31] Cushnie T., Cushnie B., Echeverría J., Fowsantear W., Thammawat S., Dodgson J.L., Law S., Clow S.M. (2020). Bioprospecting for antibacterial drugs: A multidisciplinary perspective on natural product source material, bioassay selection and avoidable pitfalls. Pharm. Res..

[32] Kshirsagar M.M., Dodamani A.S., Vishwakarma P., Mali G., Khobragade V.R., Deokar R.N. (2021). Comparative Assessment of Antibacterial Efficacy of Commercially Available Different Dental Gels: An In-vitro Study. Rev. Recent Clin. Trials.

[33] Jumpathong J., Nuengchamnong N., Masin K., Nakaew N., Suphrom N. (2019). Thin layer chromatography-bioautography assay for antibacterial compounds from *Streptomyces* sp. TBRC 8912, a newly isolated actinomycin D producer. Chiang Mai J. Sci..

[34] Barale S.S., Ghane S.G., Sonawane K.D. (2022). Purification and characterization of antibacterial surfactin isoforms produced by *Bacillus velezensis* SK. AMB Express.

[35] Grzelak E.M., Hwang C., Cai G., Nam J.-W., Choules M.P., Gao W., Lankin D.C., McAlpine J.B., Mulugeta S.G., Napolitano J.G. (2016). Bioautography with TLC-MS/NMR for rapid discovery of anti-tuberculosis lead compounds from natural sources. ACS Infect. Dis..

[36] Choma I.M., Jesionek W. (2015). TLC-direct bioautography as a high throughput method for detection of antimicrobials in plants. Chromatography.

[37] Choma I.M., Grzelak E.M. (2011). Bioautography detection in thin-layer chromatography. J. Chromatogr. A.

[38] Joseph F.-J.R.S., Iniyan A.M., Vincent S.G.P. (2017). HR-LC-MS based analysis of two antibacterial metabolites from a marine sponge symbiont *Streptomyces pharmamarensis* ICN40. Microb. Pathog..

[39] Matarrita-Carranza B., Murillo-Cruz C., Avendaño R., Ríos M.I., Chavarría M., Gómez-Calvo M.L., Tamayo-Castillo G., Araya J.J., Pinto-Tomás A.A. (2021). *Streptomyces* sp. M54: An actinobacteria associated with a neotropical social wasp with high potential for antibiotic production. Antonie Van Leeuwenhoek.

[40] Chen Y., Schwack W. (2014). High-performance thin-layer chromatography screening of multi class antibiotics in animal food by bioluminescent bioautography and electrospray ionization mass spectrometry. J. Chromatogr. A.

[41] Heep J., Tuchecker P.H., Gebhardt C.R., Dürr M. (2019). Combination of thin-layer chromatography and mass spectrometry using cluster-induced desorption/ionization. ACS Omega.

[42] Kreuzig F. (1977). Application of quantitative high-performance thin-layer chromatography in the antibiotic industry. J. Chromatogr. A.

[43] Shirling E.T., Gottlieb D. (1966). Methods for characterization of *Streptomyces* species. Int. J. Syst. Bacteriol..

[44] Rajan B.M., Kannabiran K. (2014). Extraction and identification of antibacterial secondary metabolites from marine *Streptomyces sp.* VITBRK2. Int. J. Mol. Cell. Med..

[45] Wu R.-Y. (1984). Studies on the *Streptomyces* SC4. II Taxonomic and biological characteristics of *Streptomyces* strain SC4. Bot. Bull. Acad. Sin..

[46] Chaudhary H.S., Yadav J., Shrivastava A.R., Singh S., Singh A.K., Gopalan N. (2013). Antibacterial activity of actinomycetes isolated from different soil samples of Sheopur (A city of central India). J. Adv. Pharm. Technol. Res..

[47] Ganesan P., Reegan A.D., David R.H.A., Gandhi M.R., Paulraj M.G., Al-Dhabi N.A., Ignacimuthu S. (2017). Antimicrobial activity of some actinomycetes from Western Ghats of Tamil Nadu, India. Alex. J. Med..

[48] Kumar P.S., Duraipandiyan V., Ignacimuthu S. (2014). Isolation, screening and partial purification of antimicrobial antibiotics from soil *Streptomyces* sp. SCA 7. Kaohsiung J. Med. Sci..

[49] Sharma M., Manhas R.K. (2019). Purification and characterization of actinomycins from *Streptomyces* strain M7 active against methicillin resistant *Staphylococcus aureus* and vancomycin resistant *Enterococcus*. BMC Microbiol..

[50] Qureshi K.A., Bholay A.D., Rai P.K., Mohammed H.A., Khan R.A., Azam F., Jaremko M., Emwas A.-H., Stefanowicz P., Waliczek M. (2021). Isolation, characterization, anti-MRSA evaluation, and in-silico multi-target anti-microbial validations of actinomycin X2 and actinomycin D produced by novel *Streptomyces smyrnaeus* UKAQ_23. Sci. Rep..

[51] Sharma P., Kalita M.C., Thakur D. (2016). Broad spectrum antimicrobial activity of forest-derived soil actinomycete, *Nocardia sp.* PB-52. Front. Microbiol..

[52] Saravana Kumar P., Al-Dhabi N.A., Duraipandiyan V., Balachandran C., Praveen Kumar P., Ignacimuthu S. (2014). In vitro antimicrobial, antioxidant and cytotoxic properties of *Streptomyces lavendulae* strain SCA5. BMC Microbiol..

[53] Koberska M., Vesela L., Vimberg V., Lenart J., Vesela J., Kamenik Z., Janata J., Novotna G.B. (2020). Beyond self-resistance: ABCF ATPase LmrC is a signal-transducing component of an antibiotic-driven signaling cascade hastening the onset of lincomycin biosynthesis. bioRxiv.

[54] Ibrahim A.A., El-Housseiny G.S., Aboshanab K.M., Yassien M.A., Hassouna N.A. (2019). Paromomycin production from *Streptomyces rimosus* NRRL 2455: Statistical optimization and new synergistic antibiotic combinations against multidrug resistant pathogens. BMC Microbiol..

[55] Kavanagh F. (1975). Microbiological diffusion assay II: Design and applications. J. Pharm. Sci..

[56] Pudi N., Varikuti G.D., Badana A.K., Gavara M.M., Kumari S., Malla R. (2016). Studies on optimization of growth parameters for enhanced production of antibiotic alkaloids by isolated marine actinomycetes. J. Appl. Pharm. Sci..

[57] Rütten A., Wohlleben W., Mitousis L., Musiol-Kroll E.M., Sass P. (2022). A Whole-Cell Assay for Detection of Antibacterial Activity in Actinomycetes Culture Supernatants. Antibiotics: Methods in Protocols.

[58] Bonev B., Hooper J., Parisot J. (2008). Principles of assessing bacterial susceptibility to antibiotics using the agar diffusion method. J. Antimicrob. Chemother..

[59] Clinical and Laboratory Standards Institute (CLSI) (2018). Method for Antifungal Disk Diffusion Susceptibility Testing of Yeasts.

[60] Wayne P., Clinical and Laboratory Standards Institute (CLSI) (2004). M44-A2: Method for Antifungal Disk Diffusion Susceptibility Testing of Yeasts.

[61] Meyer R., Young L., Armstrong D. (1971). Tobramycin (nebramycin factor 6): In vitro activity against *Pseudomonas aeruginosa*. Appl. Microbiol..

[62] Mitousis L., Maier H., Martinovic L., Kulik A., Stockert S., Wohlleben W., Stiefel A., Musiol-Kroll E.M. (2021). Engineering of *Streptoalloteichus tenebrarius* 2444 for Sustainable Production of Tobramycin. Molecules.

[63] Hirsch H.A., Kunin C.M., Finland M., Wilcox C., Najarian A. (1959). Antibacterial activity of serum of normal men after oral doses of erythromycin propionate and triacetyloleandomycin. New Engl. J. Med..

[64] Rasmussen F. (1959). Mammary Excretion of Benzylpenicillin, Erythromycin, and Penethamate Hydroiodide*. Acta Pharmacol. Toxicol..

[65] Consden R., Gordon A.H., Martin A.J.P. (1944). Qualitative analysis of proteins: A partition chromatographic method using paper. Biochem. J..

[66] Goodall R., Levi A. (1946). A microchromatographic method for the detection and approximate determination of the different penicillins in a mixture. Nature.

[67] Fischer R., Lautner H. (1961). Zum papierchromatographischen Nachweis von Penicillinpräparaten. Arch. Pharm..

[68] Wang M., Zhang Y., Wang R., Wang Z., Yang B., Kuang H. (2021). An evolving technology that integrates classical methods with continuous technological developments: Thin-layer chromatography bioautography. Molecules.

[69] Hölzl G., Dörmann P. (2021). Thin-Layer Chromatography. Plant Lipids.

[70] Skorupa A., Gierak A. (2011). Detection and visualization methods used in thin-layer chromatography. JPC-J. Planar Chromatogr. -Mod. TLC.

[71] Aszalos A., Frost D. (1975). [8] Thin-layer chromatography of antibiotics. Methods in Enzymology.

[72] Dewanjee S., Gangopadhyay M., Bhattacharya N., Khanra R., Dua T.K. (2015). Bioautography and its scope in the field of natural product chemistry. J. Pharm. Anal..

[73] Núñez-Montero K., Lamilla C., Abanto M., Maruyama F., Jorquera M.A., Santos A., Martinez-Urtaza J., Barrientos L. (2019). Antarctic *Streptomyces fildesensis* So13. 3 strain as a promising source for antimicrobials discovery. Sci. Rep..

[74] Couillerot O., Loqman S., Toribio A., Hubert J., Gandner L., Nuzillard J.-M., Ouhdouch Y., Clément C., Barka E.A., Renault J.-H. (2014). Purification of antibiotics from the biocontrol agent *Streptomyces anulatus* S37 by centrifugal partition chromatography. J. Chromatogr. B.

[75] Sharma K., Mullangi R. (2013). A concise review of HPLC, LC-MS and LC-MS/MS methods for determination of azithromycin in various biological matrices. Biomed. Chromatogr..

[76] Wong A.L.-A., Xiang X., Ong P.S., Mitchell E.Q.Y., Syn N., Wee I., Kumar A.P., Yong W.P., Sethi G., Goh B.C. (2018). A review on liquid chromatography-tandem mass spectrometry methods for rapid quantification of oncology drugs. Pharmaceutics.

[77] De Girolamo A., Lippolis V., Pascale M. (2022). Overview of Recent Liquid Chromatography Mass Spectrometry-Based Methods for Natural Toxins Detection in Food Products. Toxins.

[78] Ito T., Masubuchi M. (2014). Dereplication of microbial extracts and related analytical technologies. J. Antibiot..

[79] Jose P.A., Jha B. (2016). New dimensions of research on actinomycetes: Quest for next generation antibiotics. Front. Microbiol..

[80] Farha M.A., Brown E.D. (2016). Strategies for target identification of antimicrobial natural products. Nat. Prod. Rep..

[81] Leshchiner D., Rosconi F., Sundaresh B., Rudmann E., Ramirez L.M.N., Nishimoto A.T., Wood S.J., Jana B., Bujan N., Li K. (2022). A genome-wide atlas of antibiotic susceptibility targets and pathways to tolerance. Nat. Commun..

[82] Emmerich C.H., Gamboa L.M., Hofmann M.C.J., Bonin-Andresen M., Arbach O., Schendel P., Gerlach B., Hempel K., Bespalov A., Dirnagl U. (2021). Improving target assessment in biomedical research: The GOT-IT recommendations. Nat. Rev. Drug Discov..

[83] Li G., Peng X., Guo Y., Gong S., Cao S., Qiu F. (2021). Currently Available Strategies for Target Identification of Bioactive Natural Products. Front. Chem..

[84] Kitagawa M., Ara T., Arifuzzaman M., Ioka-Nakamichi T., Inamoto E., Toyonaga H., Mori H. (2005). Complete set of ORF clones of Escherichia coli ASKA library (a complete set of E. coli K-12 ORF archive): Unique resources for biological research. DNA Res..

[85] Xiao Y., Gerth K., Muller R., Wall D. (2012). Myxobacterium-produced antibiotic TA (myxovirescin) inhibits type II signal peptidase. Antimicrob. Agents Chemother..

[86] Cuatrecasas P., Wilchek M., Anfinsen C.B. (1968). Selective enzyme purification by affinity chromatography. Proc. Natl. Acad. Sci. USA.

[87] Gosschalk J.E., Chang C., Sue C.K., Siegel S.D., Wu C., Kattke M.D., Yi S.W., Damoiseaux R., Jung M.E., Ton-That H. (2020). A cell-based screen in actinomyces oris to identify Sortase inhibitors. Sci. Rep..

[88] Jonkers T.J., Steenhuis M., Schalkwijk L., Luirink J., Bald D., Houtman C.J., Kool J., Lamoree M.H., Hamers T. (2020). Development of a high-throughput bioassay for screening of antibiotics in aquatic environmental samples. Sci. Total Environ..

[89] Paytubi S., de La Cruz M., Tormo J.R., Martín J., González I., Gonzalez-Menendez V., Genilloud O., Reyes F., Vicente F., Madrid C. (2017). A high-throughput screening platform of microbial natural products for the discovery of molecules with antibiofilm properties against Salmonella. Front. Microbiol..

[90] Liao J., Xu G., Mevers E.E., Clardy J., Watnick P.I. (2018). A high-throughput, whole cell assay to identify compounds active against carbapenem-resistant Klebsiella pneumoniae. PLoS ONE.

[91] Riss T.L., Moravec R.A., Niles A.L., Duellman S., Benink H.A., Worzella T.J., Minor L. (2016). Cell viability assays. Assay Guidance Manual.

[92] Qiu T.A., Nguyen T.H.T., Hudson-Smith N.V., Clement P.L., Forester D.-C., Frew H., Hang M.N., Murphy C.J., Hamers R.J., Feng Z.V. (2017). Growth-based bacterial viability assay for interference-free and high-throughput toxicity screening of nanomaterials. Anal. Chem..

[93] Kamiloglu S., Sari G., Ozdal T., Capanoglu E. (2020). Guidelines for cell viability assays. Food Front..

[94] Northrup J.D., Mach R.H., Sellmyer M.A. (2019). Radiochemical approaches to imaging bacterial infections: Intracellular versus extracellular targets. Int. J. Mol. Sci..

[95] Barbosa M.D., Yang G., Fang J., Kurilla M.G., Pompliano D.L. (2002). Development of a whole-cell assay for peptidoglycan biosynthesis inhibitors. Antimicrob. Agents Chemother..

[96] Brown A.R., Gordon R.A., Hyland S.N., Siegrist M.S., Grimes C.L. (2020). Chemical biology tools for examining the bacterial cell wall. Cell Chem. Biol..

[97] Melamed S., Lalush C., Elad T., Yagur-Kroll S., Belkin S., Pedahzur R. (2012). A bacterial reporter panel for the detection and classification of antibiotic substances. Microb. Biotechnol..

[98] Schäfer A.-B., Wenzel M. (2020). A how-to guide for mode of action analysis of antimicrobial peptides. Front. Cell. Infect. Microbiol..

[99] Yoon S.A., Park S.Y., Cha Y., Gopala L., Lee M.H. (2021). Strategies of detecting bacteria using fluorescence-based dyes. Front. Chem..

[100] Hutter B., Fischer C., Jacobi A., Schaab C., Loferer H. (2004). Panel of *Bacillus subtilis* reporter strains indicative of various modes of action. Antimicrob. Agents Chemother..

[101] Moir D.T., Di M., Opperman T., Schweizer H.P., Bowlin T.L. (2007). A high-throughput, homogeneous, bioluminescent assay for *Pseudomonas aeruginosa* gyrase inhibitors and other DNA-damaging agents. J. Biomol. Screen..

[102] Moore S.J., MacDonald J.T., Wienecke S., Ishwarbhai A., Tsipa A., Aw R., Kylilis N., Bell D.J., McClymont D.W., Jensen K. (2018). Rapid acquisition and model-based analysis of cell-free transcription–translationreactions from nonmodel bacteria. Proc. Natl. Acad. Sci. USA.

[103] Musiol-Kroll E.M., Zubeil F., Schafhauser T., Härtner T., Kulik A., McArthur J., Koryakina I., Wohlleben W., Grond S., Williams G.J. (2017). Polyketide bioderivatization using the promiscuous acyltransferase KirCII. ACS Synth. Biol..

[104] Tholander F., Sjöberg B.-M. (2012). Discovery of antimicrobial ribonucleotide reductase inhibitors by screening in microwell format. Proc. Natl. Acad. Sci. USA.

[105] Orelle C., Carlson S., Kaushal B., Almutairi M.M., Liu H., Ochabowicz A., Quan S., Pham V.C., Squires C.L., Murphy B.T. (2013). Tools for characterizing bacterial protein synthesis inhibitors. Antimicrob. Agents Chemother..

[106] Farah N., Chin V.K., Chong P.P., Lim W.F., Lim C.W., Basir R., Chang S.K., Lee T.Y. (2022). Riboflavin as a promising antimicrobial agent? A multi-perspective review. Curr. Res. Microb. Sci..

[107] Nowak M.G., Skwarecki A.S., Milewska M.J. (2021). Amino Acid Based Antimicrobial Agents—Synthesis and Properties. ChemMedChem.

[108] Magalhães J., Franko N., Raboni S., Annunziato G., Tammela P.i., Bruno A., Bettati S., Mozzarelli A., Pieroni M., Campanini B. (2020). Inhibition of nonessential bacterial targets: Discovery of a novel serine O-acetyltransferase inhibitor. ACS Med. Chem. Lett..

[109] Yao J., Rock C.O. (2017). Bacterial fatty acid metabolism in modern antibiotic discovery. Biochim. Biophys. Acta BBA-Mol. Cell Biol. Lipids.

[110] Spry C., Kirk K., Saliba K.J. (2008). Coenzyme A biosynthesis: An antimicrobial drug target. FEMS Microbiol. Rev..

[111] Collins S.M., Brown A.C. (2021). Bacterial Outer Membrane Vesicles as Antibiotic Delivery Vehicles. Front. Immunol..

[112] Uddin M.J., Dawan J., Jeon G., Yu T., He X., Ahn J. (2020). The Role of Bacterial Membrane Vesicles in the Dissemination of Antibiotic Resistance and as Promising Carriers for Therapeutic Agent Delivery. Microorganisms.

[113] Sousa M.C. (2019). New antibiotics target bacterial envelope. Nature.

[114] Caruana J.C., Walper S.A. (2020). Bacterial membrane vesicles as mediators of microbe–microbe and microbe–host community interactions. Front. Microbiol..

[115] Silbert L., Ben Shlush I., Israel E., Porgador A., Kolusheva S., Jelinek R. (2006). Rapid chromatic detection of bacteria by use of a new biomimetic polymer sensor. Appl. Environ. Microbiol..

[116] Silhavy T.J., Kahne D., Walker S. (2010). The bacterial cell envelope. Cold Spring Harb. Perspect. Biol..

[117] Pasquina-Lemonche L., Burns J., Turner R., Kumar S., Tank R., Mullin N., Wilson J., Chakrabarti B., Bullough P., Foster S. (2020). The architecture of the Gram-positive bacterial cell wall. Nature.

[118] Scheffers D.-J., Pinho M.G. (2005). Bacterial cell wall synthesis: New insights from localization studies. Microbiol. Mol. Biol. Rev..

[119] Fisher J.F., Mobashery S. (2020). Constructing and deconstructing the bacterial cell wall. Protein Sci..

[120] Dik D.A., Fisher J.F., Mobashery S. (2018). Cell-wall recycling of the Gram-negative bacteria and the nexus to antibiotic resistance. Chem. Rev..

[121] Mayer C., Kluj R.M., Muehleck M., Walter A., Unsleber S., Hottmann I., Borisova M. (2019). Bacteria’s different ways to recycle their own cell wall. Int. J. Med. Microbiol..

[122] Reyes-Lamothe R., Sherratt D.J. (2019). The bacterial cell cycle, chromosome inheritance and cell growth. Nat. Rev. Microbiol..

[123] Wang J.D., Levin P.A. (2009). Metabolism, cell growth and the bacterial cell cycle. Nat. Rev. Microbiol..

[124] Jones S. (2017). Permeability rules for antibiotic design. Nat. Biotechnol..

[125] Schuerholz T., Domming S., Hornef M., Dupont A., Kowalski I., Kaconis Y., Heinbockel L., Andra J., Garidel P., Gutsmann T. (2012). Bacterial cell wall compounds as promising targets of antimicrobial agents II. Immunological and clinical aspects. Curr. Drug Targets.

[126] Müller A., Klöckner A., Schneider T. (2017). Targeting a cell wall biosynthesis hot spot. Nat. Prod. Rep..

[127] Gram C. (1884). The differential staining of Schizomycetes in tissue sections and in dried preparations. Fortschitte Med..

[128] Beveridge T.J. (1999). Structures of gram-negative cell walls and their derived membrane vesicles. J. Bacteriol..

[129] Mehravar M., Sardari S., Owlia P. (2010). Screening of membrane active antimicrobial metabolites produced by soil actinomycetes using membrane models. J. Paramed. Sci..

[130] Reece R., Maxwell A. (1991). Critical Rev. Biochem. Mol. Biol..

[131] Chitra S., Ramalakshmi N., Arunkumar S., Manimegalai P. (2020). A comprehensive review on DNA gyrase inhibitors. Former. Curr. Drug Targets-Infect. Disord..

[132] Man R.-J., Zhang X.-P., Yang Y.-S., Jiang A.-Q., Zhu H.-L. (2021). Recent progress in small molecular inhibitors of DNA Gyrase. Curr. Med. Chem..

[133] Maxwell A., Bush N.G., Evans-Roberts K. (2015). DNA topoisomerases. EcoSal Plus.

[134] Finegold S., Miller L., Posnick D., Patterson D., Davis A. (1966). Nalidixic acid: Clinical and laboratory studies. Antimicrob. Agents Chemother..

[135] Bush N.G., Diez-Santos I., Abbott L.R., Maxwell A. (2020). Quinolones: Mechanism, lethality and their contributions to antibiotic resistance. Molecules.

[136] Torrents Serra E. (2014). Ribonucleotide reductases: Essential enzymes for bacterial life. Front. Cell. Infect. Microbiol..

[137] Ruskoski T.B., Boal A.K. (2021). The periodic table of ribonucleotide reductases. J. Biol. Chem..

[138] Borovok I., Gorovitz B., Schreiber R., Aharonowitz Y., Cohen G. (2006). Coenzyme B12 controls transcription of the *Streptomyces* class Ia ribonucleotide reductase nrdABS operon via a riboswitch mechanism. J. Bacteriol..

[139] Sergiev P.V., Komarova E.S., Osterman I.A., Pletnev P.I., Golovina A.Y., Laptev I.G., Evfratov S.A., Marusich E.I., Veselov M.S., Leonov S.V. (2018). Overview of 17,856 Compound Screening for Translation Inhibition and DNA Damage in Bacteria. Proceedings of the Scientific-Practical Conference: Research and Development-2016.

[140] Raneri M., Sciandrone B., Briani F. (2015). A whole-cell assay for specific inhibitors of translation initiation in bacteria. J. Biomol. Screen..

[141] Ramadoss N.S., Alumasa J.N., Cheng L., Wang Y., Li S., Chambers B.S., Chang H., Chatterjee A.K., Brinker A., Engels I.H. (2013). Small molecule inhibitors of trans-translation have broad-spectrum antibiotic activity. Proc. Natl. Acad. Sci. USA.

[142] Lowell A.N., Santoro N., Swaney S.M., McQuade T.J., Schultz P.J., Larsen M.J., Sherman D.H. (2015). Microscale Adaptation of In Vitro Transcription/Translation for High-Throughput Screening of Natural Product Extract Libraries. Chem. Biol. Drug Des..

[143] Montgomery J.I., Smith J.F., Tomaras A.P., Zaniewski R., McPherson C.J., McAllister L.A., Hartman-Neumann S., Arcari J.T., Lescoe M., Gutierrez J. (2015). Discovery and characterization of a novel class of pyrazolopyrimidinedione tRNA synthesis inhibitors. J. Antibiot..

[144] Brandi L., Dresios J., Gualerzi C.O. (2008). Assays for the identification of inhibitors targeting specific translational steps. New Antibiotic Targets.

[145] Kavčič B., Tkačik G., Bollenbach T. (2020). Mechanisms of drug interactions between translation-inhibiting antibiotics. Nat. Commun..

[146] Galluzzi L., Karp M. (2003). Amplified detection of transcriptional and translational inhibitors in bioluminescent *Escherichia coli* K-12. SLAS Discov..

[147] Bianchi A.A., Baneyx F. (1999). Stress responses as a tool to detect and characterize the mode of action of antibacterial agents. Appl. Environ. Microbiol..

[148] Kurittu J., Karp M., Korpela M. (2000). Detection of tetracyclines with luminescent bacterial strains. Lumin. J. Biol. Chem. Lumin..

[149] Möhrle V., Stadler M., Eberz G. (2007). Biosensor-guided screening for macrolides. Anal. Bioanal. Chem..

[150] Bailey M., Chettiath T., Mankin A.S. (2008). Induction of erm (C) expression by noninducing antibiotics. Antimicrob. Agents Chemother..

[151] Osterman I.A., Prokhorova I.V., Sysoev V.O., Boykova Y.V., Efremenkova O.V., Svetlov M.S., Kolb V.A., Bogdanov A.A., Sergiev P.V., Dontsova O.A. (2012). Attenuation-based dual-fluorescent-protein reporter for screening translation inhibitors. Antimicrob. Agents Chemother..

[152] Urban A., Eckermann S., Fast B., Metzger S., Gehling M., Ziegelbauer K., Rübsamen-Waigmann H., Freiberg C. (2007). Novel whole-cell antibiotic biosensors for compound discovery. Appl. Environ. Microbiol..

[153] Goh E.-B., Yim G., Tsui W., McClure J., Surette M.G., Davies J. (2002). Transcriptional modulation of bacterial gene expression by subinhibitory concentrations of antibiotics. Proc. Natl. Acad. Sci. USA.

[154] Svetlov M.S., Kommer A., Kolb V.A., Spirin A.S. (2006). Effective cotranslational folding of firefly luciferase without chaperones of the Hsp70 family. Protein Sci..

[155] Shimizu Y., Inoue A., Tomari Y., Suzuki T., Yokogawa T., Nishikawa K., Ueda T. (2001). Cell-free translation reconstituted with purified components. Nat. Biotechnol..

[156] Polacek N., Swaney S., Shinabarger D., Mankin A.S. (2002). SPARK A Novel Method To Monitor Ribosomal Peptidyl Transferase Activity. Biochemistry.

[157] Llano-Sotelo B., Hickerson R.P., Lancaster L., Noller H.F., Mankin A.S. (2009). Fluorescently labeled ribosomes as a tool for analyzing antibiotic binding. Rna.

[158] Nonejuie P., Burkart M., Pogliano K., Pogliano J. (2013). Bacterial cytological profiling rapidly identifies the cellular pathways targeted by antibacterial molecules. Proc. Natl. Acad. Sci. USA.

[159] Bandow J.E., BrÖtz H., Leichert L.I.O., Labischinski H., Hecker M. (2003). Proteomic approach to understanding antibiotic action. Antimicrob. Agents Chemother..

[160] Osterman I.A., Ustinov A.V., Evdokimov D.V., Korshun V.A., Sergiev P.V., Serebryakova M.V., Demina I.A., Galyamina M.A., Govorun V.M., Dontsova O.A. (2013). A nascent proteome study combining click chemistry with 2 DE. Proteomics.

[161] Osterman I.A., Bogdanov A.A., Dontsova O.A., Sergiev P.V. (2016). Techniques for screening translation inhibitors. Antibiotics.

[162] Hoagland M.B., Stephenson M.L., Scott J.F., Hecht L.I., Zamecnik P.C. (1958). A soluble ribonucleic acid intermediate in protein synthesis. J. Biol. Chem..

[163] Pratt J.M. (1984). Coupled transcription-translation in prokaryotic cell-free systems. Transcription and Translation: A Practical Approach.

[164] Hames B.D., Higgins S.J., Higgins S.J. (1984). Transcription and Translation: A Practical Approach.

[165] Kim T.-W., Keum J.-W., Oh I.-S., Choi C.-Y., Park C.-G., Kim D.-M. (2006). Simple procedures for the construction of a robust and cost-effective cell-free protein synthesis system. J. Biotechnol..

[166] Wolf H., Chinali G., Parmeggiani A. (1974). Kirromycin, an inhibitor of protein biosynthesis that acts on elongation factor Tu. Proc. Natl. Acad. Sci. USA.

[167] Wolf H., Chinali G., Parmeggiani A. (1977). Mechanism of the Inhibition of Protein Synthesis by Kirromycin: Role of Elongation Factor Tu and Ribosomes. Eur. J. Biochem..

[168] Liu X., Chen Y., Fierke C.A. (2014). A real-time fluorescence polarization activity assay to screen for inhibitors of bacterial ribonuclease P. Nucleic Acids Res..

[169] Penchovsky R., Stoilova C.C. (2013). Riboswitch-based antibacterial drug discovery using high-throughput screening methods. Expert Opin. Drug Discov..

[170] Deigan K.E., FerrÉ-D’AmarÉ A.R. (2011). Riboswitches: Discovery of drugs that target bacterial gene-regulatory RNAs. Acc. Chem. Res..

[171] Lünse C.E., Mayer G. (2017). Reporter gene-based screening for TPP riboswitch activators. Antibiotics.

[172] Breaker R.R. (2011). Prospects for riboswitch discovery and analysis. Mol. Cell.

[173] Mironov A.S., Gusarov I., Rafikov R., Lopez L.E., Shatalin K., Kreneva R.A., Perumov D.A., Nudler E. (2002). Sensing small molecules by nascent RNA: A mechanism to control transcription in bacteria. Cell.

[174] Nahvi A., Sudarsan N., Ebert M.S., Zou X., Brown K.L., Breaker R.R. (2002). Genetic control by a metabolite binding mRNA. Chem. Biol..

[175] Panchal V., Brenk R. (2021). Riboswitches as drug targets for antibiotics. Antibiotics.

[176] Kirchner M., Schorpp K., Hadian K., Schneider S. (2017). An in vivo high-throughput screening for riboswitch ligands using a reverse reporter gene system. Sci. Rep..

[177] Lee E.R., Blount K.F., Breaker R.R. (2009). Roseoflavin is a natural antibacterial compound that binds to FMN riboswitches and regulates gene expression. RNA Biol..

[178] Pedrolli D.B., Matern A., Wang J., Ester M., Siedler K., Breaker R., Mack M. (2012). A highly specialized flavin mononucleotide riboswitch responds differently to similar ligands and confers roseoflavin resistance to *Streptomyces davawensis*. Nucleic Acids Res..

[179] Pedrolli D.B., Kühm C., Sévin D.C., Vockenhuber M.P., Sauer U., Suess B., Mack M. (2015). A dual control mechanism synchronizes riboflavin and sulphur metabolism in *Bacillus subtilis*. Proc. Natl. Acad. Sci. USA.

[180] Wang H., Mann P.A., Xiao L., Gill C., Galgoci A.M., Howe J.A., Villafania A., Barbieri C.M., Malinverni J.C., Sher X. (2017). Dual-targeting small-molecule inhibitors of the *Staphylococcus aureus* FMN riboswitch disrupt riboflavin homeostasis in an infectious setting. Cell Chem. Biol..

[181] Larsson D., Flach C.-F. (2022). Antibiotic resistance in the environment. Nat. Rev. Microbiol..

[182] Du Toit A. (2022). The cost of resistance. Nat. Rev. Microbiol..

[183] Neu H.C. (1992). The crisis in antibiotic resistance. Science.

[184] Peterson E., Kaur P. (2018). Antibiotic resistance mechanisms in bacteria: Relationships between resistance determinants of antibiotic producers, environmental bacteria, and clinical pathogens. Front. Microbiol..

[185] Miethke M., Pieroni M., Weber T., Brönstrup M., Hammann P., Halby L., Arimondo P.B., Glaser P., Aigle B., Bode H.B. (2021). Towards the sustainable discovery and development of new antibiotics. Nat. Rev. Chem..

[186] Mansjö M., Johansson J. (2011). The Riboflavin analog Roseoflavin targets an FMN-riboswitch and blocks Listeria monocytogene s growth, but also stimulates virulence gene-expression and infection. RNA Biol..

[187] Champney W.S. (2008). Three methods to assay inhibitors of ribosomal subunit assembly. New Antibiotic Targets.

[188] Nikolay R., Schloemer R., Mueller S., Deuerling E. (2015). Fluorescence-based monitoring of ribosome assembly landscapes. BMC Mol. Biol..

[189] Yassin A., Mankin A.S. (2007). Potential new antibiotic sites in the ribosome revealed by deleterious mutations in RNA of the large ribosomal subunit. J. Biol. Chem..

[190] Champney W.S. (2020). Antibiotics targeting bacterial ribosomal subunit biogenesis. J. Antimicrob. Chemother..

[191] Yassin A., Fredrick K., Mankin A.S. (2005). Deleterious mutations in small subunit ribosomal RNA identify functional sites and potential targets for antibiotics. Proc. Natl. Acad. Sci. USA.

[192] Poehlsgaard J., Douthwaite S. (2005). The bacterial ribosome as a target for antibiotics. Nat. Rev. Microbiol..

[193] Blanchard S.C., Cooperman B.S., Wilson D.N. (2010). Probing translation with small-molecule inhibitors. Chem. Biol..

[194] Bem A.E., Velikova N., Pellicer M.T., Baarlen P.v., Marina A., Wells J.M. (2015). Bacterial histidine kinases as novel antibacterial drug targets. ACS Chem. Biol..

[195] Fihn C.A., Carlson E.E. (2021). Targeting a highly conserved domain in bacterial histidine kinases to generate inhibitors with broad spectrum activity. Curr. Opin. Microbiol..

[196] Chen H., Yu C., Wu H., Li G., Li C., Hong W., Yang X., Wang H., You X. (2022). Recent Advances in Histidine Kinase-Targeted Antimicrobial Agents. Front. Chem..

[197] Boyle-Vavra S., Yin S., Daum R.S. (2006). The VraS/VraR two-component regulatory system required for oxacillin resistance in community-acquired methicillin-resistant *Staphylococcus aureus*. FEMS Microbiol. Lett..

[198] Kinoshita-Kikuta E., Maruta S., Eguchi Y., Igarashi M., Okajima T., Utsumi R., Kinoshita E., Koike T. (2020). An immuno-dot blot assay for screening histidine kinase inhibitors. Anal. Biochem..

[199] Marina A., Mott C., Auyzenberg A., Hendrickson W.A., Waldburger C.D. (2001). Structural and mutational analysis of the PhoQ histidine kinase catalytic domain: Insight into the reaction mechanism. J. Biol. Chem..

[200] Velikova N., Fulle S., Manso A.S., Mechkarska M., Finn P., Conlon J.M., Oggioni M.R., Wells J.M., Marina A. (2016). Putative histidine kinase inhibitors with antibacterial effect against multi-drug resistant clinical isolates identified by in vitro and in silico screens. Sci. Rep..

[201] Ma P., Phillips-Jones M.K. (2021). Membrane sensor histidine kinases: Insights from structural, ligand and inhibitor studies of full-length proteins and signalling domains for antibiotic discovery. Molecules.

[202] Guarnieri M.T., Blagg B.S., Zhao R. (2011). A high-throughput TNP-ATP displacement assay for screening inhibitors of ATP-binding in bacterial histidine kinases. Assay Drug Dev. Technol..

[203] Zhang L., Quan C., Zhang X., Xiong W., Fan S. (2019). Proteoliposome-based model for screening inhibitors targeting histidine kinase AgrC. Chem. Biol. Drug Des..

[204] Li N., Wang F., Niu S., Cao J., Wu K., Li Y., Yin N., Zhang X., Zhu W., Yin Y. (2009). Discovery of novel inhibitors of *Streptococcus pneumoniae* based on the virtual screening with the homology-modeled structure of histidine kinase (VicK). BMC Microbiol..

[205] Wilke K.E., Francis S., Carlson E.E. (2015). Inactivation of multiple bacterial histidine kinases by targeting the ATP-binding domain. ACS Chem. Biol..

[206] King A., Blackledge M.S. (2021). Evaluation of small molecule kinase inhibitors as novel antimicrobial and antibiofilm agents. Chem. Biol. Drug Des..

[207] World Health Organization (WHO) Antibiotic Resistance. https://www.who.int/news-room/fact-sheets/detail/antibiotic-resistance.

[208] Cheng G., Dai M., Ahmed S., Hao H., Wang X., Yuan Z. (2016). Antimicrobial drugs in fighting against antimicrobial resistance. Front. Microbiol..

[209] Kohanski M.A., Dwyer D.J., Collins J.J. (2010). How antibiotics kill bacteria: From targets to networks. Nat. Rev. Microbiol..

[210] Cook M.A., Wright G.D. (2022). The past, present, and future of antibiotics. Sci. Transl. Med..

[211] Hudson M.A., Lockless S.W. (2022). Elucidating the Mechanisms of Action of Antimicrobial Agents. Mbio.

[212] Belete T.M. (2019). Novel targets to develop new antibacterial agents and novel alternatives to antibacterial agents. Hum. Microbiome J..

[213] Tondi D. (2021). Novel targets and mechanisms in antimicrobial drug discovery. Antibiotics.

[214] Vincent I.M., Ehmann D.E., Mills S.D., Perros M., Barrett M.P. (2016). Untargeted Metabolomics To Ascertain Antibiotic Modes of Action. Antimicrob. Agents Chemother..

